# Some Drug Experiences

**Published:** 1899-12

**Authors:** A. G. Downer

**Affiliations:** Princeton, Ill.


### SOME DRUG EXPERIENCES.

A. G. Downer, M. D., Princeton, Ill.

The manner of teaching students the art and method of prescribing is all wrong; that is generally speaking, as any physician who is a student, after a practice of ten years must agree with me. I graduated from the New York Homœopathic Medical College, taking the three years' course, and I am fully satisfied with the course in general, but the instruction in materia medica was not satisfactory. Why? To begin every drug has its own particular individuality and characteristics, which in the main are easily and quickly comprehended. Then what? Next relationships, conditions of aggravation, amelioration, but these points are only lightly touched upon, and let me say here, I never heard a word said about the miasms, psora or sycosis, etc. Then again the question of potency was utterly ignored, here of course we begin to tread on the only debatable ground that has been fought over and over again. One says, tinctures to 3x dilutions or triturations. No, says another, 30x to 200x. Utterly wrong, says the high potency man, 200x to 1 M. to C. M. always, and a single dose. From my experience all are right, but none altogether so. However, the closer the man prescribes and that a high potency, and has the courage to wait, that man will attain the best and lasting results. I well know we must cause terrible aggravations by low prescribing, and why not, if our grand law of cure is true?

As before said, when in college never in lecture or clinic did I hear a high potency prescribed, except from the late

Samuel Lillienthal or Dr. Burdick. And never once heard a lecture on the use of high potencies, and worse yet, left college thinking high potencies and their users only visionary, theoretic cranks. And was it not natural?

I honor the position Dunham College of Chicago has taken to teach pure Homœopathy, and the use of the high potencies and the unadulterated law of cure as laid down, line upon line by father Hahnemann.

It is not strange that so many good would-be students degenerate into mongrels, and the question comes, why is this? My idea is the prescription is, perhaps, good enough, but too often too pat to the case, and naturally an aggravation is caused. Why should it not? Is the law true or false? And instead of waiting or going higher a change is made, the case is mixed up; patient and physican are disgusted; the physician resorts to some mongrel palliative and some way the patient crawls from under the load of drug and disease, and the good doctor drifts and drifts and is gone. Another reason is a chronic indisposition to exertion to study the cases. It takes courage, my fellow-physicians, to step out on the high potency premises and still more faith and courage to wait and see the salvation which comes.

Drug aggravations cause a new disease on the base of the original one, and are the hardest to prescribe for, and why not? I am deeply indebted to Dr. E. W. Sawyer, of Chicago, for a revelation in medicine in the high potencies, the antidotal relations of high for the low, and his interpretations of points of interest of syphilis, psora, and sycosis. I am only slowly creeping now, but fully hope some day to rise and walk with full confidence in the light.

My first experience was with Silicea, throwing the 3x bottle out of the window and using the 30x. I can see you smile at my boldness, but the 30x did far better than the 3x, and now I can pin my faith on Silicea anywhere from 30x to C. M., and know it is faithful to the trust I give it. And my trial on other drugs was continued in the same manner. I find I am not alone in my search after light, recently in conversation with a successful homœopathic physican of thirty-five years experience and who stands high in the profession and his native city, but this man had no experiency of any potency higher than 30x and that rarely, and knew nothing of the homœopathic antidotal drug relation, and nothing of the nosodes. Just think what this man has missed by not knowing anything about these points. Why, I think my most brilliant successes have been made by simply antidoting drug effects, lifting off a burden of drugs greater by far to bear than the disease itself. My first two cases were just to lift a load of Quinine off the sufferer with a high potency of Quinine, and the first case only took one dose for an ailment of ten years' standing. The second case was one in which the antidotal dose of Quinine high had to be repeated three times. I am telling no fairy tales. All I say, try for yourself.

Not enough stress is laid on conditions of aggravation and amelioration, and why should this not be? What makes worse, what makes better, but not a word in college lectures. The professor comes in and fills up his hour with a book lecture of symptoms from A to Z, anything a student can find out for himself, but the real point of the drug is not touched upon. To be sure the study of materia medica is a matter of each individual who studies his case from the foundation. Some things no one can learn for you, but to direct the student into the right line of study is the thing. Teachers are supposed to direct, aid, assist. Any one can take Hering, Lippe, or any text-book and give a lecture, but that is not wholly the point.

In the April *Medical Advance*, page 280, a physician, Dr. J. W. Cartlich, comes out flat-footed and withdraws from the Kansas City Homœopathic Medical College, giving as his reasons: First. That the teaching is wrong; advising alternation, mixing *one*, *two* or three remedies together. Second.

Ridicule of single remedy and high potency. Third. Graduating any who apply. I honor such a man. The Allopathic school still clings to its grand triumvirate of drugs, Morphine, Quinine and Whisky. These are surely by this time canonized allopathic gods.

Hahnemann's *Chronic Diseases* is a powerful, masterful production appealing to reason. Clear, clean-cut, concise, and, from my experience, it is true. Read it and *The Organon* and then read the shallow, vacillating, shifting, experimental subterfuge of Allopathic methods, the more you read the less you are sure of, and ends in—oh, well, an anodyne—or some makeshift. Verily "A diarrhœa of words and a constipation of ideas."

I have found by first antidoting the drug symptoms, which are masking the case, a great strategic plan. A lady wrote me from Iowa, a letter containing thirteen pages of woe. The facts were, her whole left side had been decorated with Iodine for a heart trouble, after they had used their slender means to relieve her and she came very near being ruined by the Iodine. I at once gave her one dose Iodine C. M. and made no other prescription for a month; but let me say the Iodine C. M. lifted off the burden she was carrying and began at once to improve. Can anything act better? The lady wrote saying: "Why couldn't these physicians help me, seeing me every day, and you send me a little medicine which helps me at once?"

Vaccination to-day is a ruinous, pernicious practice, and I have not a bit of doubt that many people are ruined yearly. And here the high potencies are effective in antidoting the crude drug effect, and its consequent train of symptoms, besides this I have given the C. M. potency of Variolinum and had all the symptoms of the crude vaccination itself, backache, malaise, fever, minus the awful consequences which are usually attendant upon the common method.

It is time, fully time, that widespread attention be given to these truths, first to students, and second to physicians who know they do not grasp fully the true spirit of the law. And some broad liberal means should be sought and enacted to educate the masses to grasp and avail themselves of the advantages of homœopathic practice. I do not think homœopathic physicians are outspoken enough for the cause. Allopathy is continually flaunting, with flaming advertisement, their false creed, and with jealous, spiteful invective and ridicule assailing the grand law of cure, which they cannot and will not understand. Then let us more openly proclaim our wonderful law of cure. Of course the way to down them is to do so by showing the difference everywhere, and this is continually being done; but some way the masses of people, the common people, do not fully appreciate its great value. The start must come from physicians themselves, and, as I have before stated, the right education given to their students, then when these students commence they will commence right. They will not feel as I did, after ten years of practice, that they had been ten years groping around in the dark. Our medical schools need a *heroic* purging of mongrelism. I also think the press should be used as an educator. Say, for instance, some popular Chicago daily once a week publish a column article from the pen of some well-known homœopathist, relative to Homœopathy. This would be a plan to attract attention and would result in much good, and I recommend this to the committee on publication for their action. Of course we are a busy people and comparatively few in numbers.

Homœopathy has come to stay. We must not allow her to degenerate. We should have one of the insane asylums under our control in Illinois. Why are we so behind? We have all the truth on our side and should take advantage of what we have. But materia medica must be taught right; it is the keystone of the arch, and I plead for a higher education of our students.—*The Medical Visitor*.

